# Influences for Gender Disparity in Academic Family Medicine in North American Medical Schools

**DOI:** 10.7759/cureus.8368

**Published:** 2020-05-30

**Authors:** Szu-Yu Tina Chen, Sabeena Jalal, Maryam Ahmadi, Kiran Khurshid, Nizar Bhulani, Ateeq U Rehman, Aftab Ahmad, Jeffrey Ding, Terri-Leigh R Aldred, Faisal Khosa

**Affiliations:** 1 Family Medicine, University of British Columbia, Vancouver, CAN; 2 Radiology, Vancouver General Hospital, Vancouver, CAN; 3 Surgery, Brigham and Women’s Hospital, Boston, USA; 4 Internal Medicine, Marshfield Clinic Health System, Marshfield, USA; 5 Internal Medicine, Mercer University School of Medicine, Macon, USA; 6 Internal Medicine, Orange Park Medical Center, Orange Park, USA; 7 Medicine, University of British Columbia, Vancouver, CAN; 8 Family Medicine, University of British Columbia, Prince George, CAN

**Keywords:** north america, canada, usa, gender disparity, family medicine, leadership, academic position, productivity, h-index

## Abstract

Background

Women physicians continue to comprise the minority of leadership roles in Academic Family Medicine (AFM) faculty across North American medical schools. Our study quantified the current state of gender disparity by analyzing academic position, leadership ranking, and research productivity.

Methods

We generated a database for 6,746 AFM faculty members. Gender and academic profiles were obtained for 2,892 academic ranks and 1,706 leadership roles by searching faculty listings enlisted in Fellowship and Residency Electronic Interactive Database (FREIDA) and Canadian Resident Matching Service (CaRMS). To measure research productivity, we obtained bibliometric data: h-index, citations, and tenure from 2,383 faculty members using Elsevier’s SCOPUS archives. Data analysis and h-index were formulated using Stata version 14.2 (StataCorp LP, College Station, TX).

Results

Our results indicated that women hold 46.11% (3,110/6,746) of faculty positions. The proportional composition decreased with increasing academic ranking (49.84% assistant, 46.78% associate, and 41.5% full professor). The same decreasing trend was demonstrated with leadership rank (57.14% minor leadership, 47.65% second-in-command, and 36.61 first-in-command). Compared to their gender counterparts, women in AFM demonstrated lower publication productivity as measured by citation number (p=0.04) and years of study (p=0.008). The final prediction equation model after multivariable analyses included gender, publications, citations, country of graduation, and years of active research (p<0.05).

Conclusions

The composition of academic family medicine faculty members included in this study demonstrated gender disparity. Inclusivity initiatives and policies to tackle the issue of female retention, promotion, and recruitment need to be further explored.

## Introduction

Women are entering North American medical schools in greater numbers compared to their male counterparts [1]. Academic physicians participate in scholarly engagements versus non-academic physicians who primarily focus on clinical practice. The nature of primary care practice places Academic Family Medicine (AFM) in the role of providing the first-line contact for many minority populations [2]. With awareness of gender equality, AFM leads in diversity of medical school faculty with fourfold growth of 1,396 positions held by women 30 years ago to 5,507 positions in 2015 [3]. Despite these changes, the Association of American Medical Colleges (AAMC) published that only 39.5% of faculty positions in US medical schools were held by women in 2016 [4].

This gap is not just in faculty composition. Women continue to occupy lower-ranking faculty positions such as assistant professorships and therefore obtain fewer positions of full professorships [3]. Under-representation of women in faculty composition and leadership roles is described as gender disparity [5]. This disparity has been documented in medical and surgical specialties, editorial boards, professional societies, and authorships [6-10]. It contributes to the current lack of advancement, career satisfaction, and professional confidence in women in academic medicine [11]. In addition, a paucity of role models influences medical student choice of school for matriculation and future diversity in academic positions [12]. The implications of this gap in representation in health practices and research ultimately result in ineffective use of women’s qualifications and decreased quality of care as seen in the context of increased mortality [13,14].

Once medical faculty members engage in their roles, they inherit multiple overlapping and synergistic responsibilities that have implications on funding, promotion of rank, and appointment to leadership positions. An individual's research productivity can be a major deciding factor for academic milestones, which are assessed using complementary factors such as research publications, citations, and years of research. Notably, the Hirsch (h) index is an author-level single-number metric of research performance that measures both the set of the author’s most cited papers and the number of citations their publications have received to quantify the impact of a researcher’s academic productivity through publications [15]. Analysis of research productivity in family medicine faculty members is scarce, and so the premise of our study is to shed some light on research output in AFM [16].

According to our literature search, no prior study has utilized the h-index to provide a prediction model that takes into consideration factors that impact the h-index of women in AFM while adjusting for gender disparity against confounders. We hope that creating a model will assist researchers in identifying factors leading to gender disparity in academic productivity in AFM. The aim of our retrospective cross-sectional study was to provide insight into the gender disparity among AFM healthcare providers using objective, non-self-reported data.

## Materials and methods

Data collection

We collected cross-sectional data for 6,746 AFM and administrative faculty members across the US and Canada. Data collection occurred between April and June 2017. The Fellowship and Residency Electronic Interactive Database (FREIDA) was used to obtain a list of Accreditation Council for Graduate Medical Education (ACGME) accredited Family Medicine Programs in the US. The Canadian Resident Matching Service (CaRMS) website was used to obtain information on AFM faculty members in Canadian family medicine programs. Our sources for faculty positions and ranks were obtained from respective program websites. We excluded any programs that did not have faculty listings. North American or International Medical Graduate data were obtained from program websites, FRIEDA, and CaRMS. Institutional review board approval was not required for this study because all data used were acquired from publicly available sources.

Gender Identification

The gender of all members was identified in a systematic manner. We searched for gender information found within biographies provided by the faculty members’ university website, department website, LinkedIn, ResearchGate, and/or Doximity. If we were not able to identify gender information after conducting an exhaustive search, then the faculty member was excluded.

Rank

The inclusion criteria included full-time faculty members with the academic ranking of assistant professor, associate professor, and full professor. Furthermore, faculty members were included only if they had an MD degree or equivalent (e.g. DO, MBBS, MBChB) and a listing on their university website. The faculty with departmental leadership roles included first-in-command (heads, chief, chairperson, and program directors), and second-in-command (vice chair, associate directors and deputy director). All other positions were labeled as "minor" leadership positions, which included positions that reported to someone that did not fall under any of the first- or second-in-command positions. The following were excluded: faculty with no academic ranks, adjunct and retired faculty, and faculty without an MD degree.

Productivity

Citation, publication, h-index, and data for years of active research were available for 2,383 faculty members. Elsevier’s SCOPUS citation database was used to gather data pertaining to publications, h-index, citations, and tenure of the productivity of each faculty member. The years of active research data recorded as the publication range from the first to the most recent article. Among the major bibliometric databases (Google Scholar, SCOPUS and Web of Science), we chose to utilize SCOPUS due to its high growth rates in the average number of papers, citations, and h-indices compared to its counterparts [17]. SCOPUS has 40 million publications recorded and is a reliable tool for calculating h-index because of its ability to distinguish authors [18].

Statistical analysis

Frequency and percentages were reported for categorical variables. Medians and ranges were reported for continuous variables, as the data did not follow the normal distribution. Data were checked for normality using the Kolmogorov-Smirnov test and histograms. The Mann-Whitney U test was applied to see the difference between men and women faculty members' academic productivity. The Kruskal-Wallis test was applied to see the difference in the same continuous variables among the different academic ranks. Log transformation was utilized to analyze the continuous variables of h indices, citations, and publications.

At the univariate level, simple linear regression was applied. Each variable was regressed independently with h indices, their assumptions were checked, and their significance was identified. The primary outcome was academic productivity. Independent variables included: gender, publications, citations, academic ranks, and leadership ranks. P values were calculated for univariate level analysis set at 0.25 and multivariable level analysis at 0.05. Step forward technique was used for model building.

Each of these was then selected for inclusion into multivariable linear regression analysis. The correlation coefficient was used to analyze multicollinearity between independent variables, with 0.8 being treated as the presence of multicollinearity. Cramer’s V test was used for one nominal variable and one ordinal variable. Spearman's test was used for one continuous variable and one ordinal variable. Determination for inclusion in the model was identified using a stepwise selection strategy, and p values were analyzed. The size of the F test was used to form the model. Once the preliminary model was created, we checked for interactions between each of the included main effects in the model using a cutoff set at 0.1. All data analyses were done using STATA version 14.2 (StataCorp LP, College Station, TX).

## Results

Characteristics of the faculty sample

A total of 6,746 family medicine faculty members met the inclusion criteria: there were 3,636 (53.9%) and 3,110 (46.1%) male and female members, respectively (Table 1).

**Table 1:** Total number of family medicine faculty members by academic rank and leadership position in North America First-in-Command: Heads, Chiefs, Chairperson, and Program Directors. Second-in-Command: Associate Directors & Associate/Deputy/Vice Chairperson. Minor Leadership Position: All other positions were labeled as "minor" leadership positions, which included positions that reported to someone that did not fall under any of the first- or second-in-command positions.

Variables	Males	Females
Total Number (%)	3636 (53.9%)	3110 (46.1%)
Academic Rank		
Assistant Professor	953 (50.2%)	947 (49.8%)
Associate Professor	306 (53.2%)	269 (46.8%)
Professor	241 (58.5%)	171 (41.5%)
Total	1500 (52.0%)	1387 (48.0%)
Leadership		
First-in-Command	639 (63.4%)	369 (36.6%)
Second-in-Command	356 (52.4%)	324 (47.6%)
Minor Leadership Position	6 (42.9%)	8 (57.1%)
Total	1001 (58.8%)	701 (41.2%)

**Figure 1:**
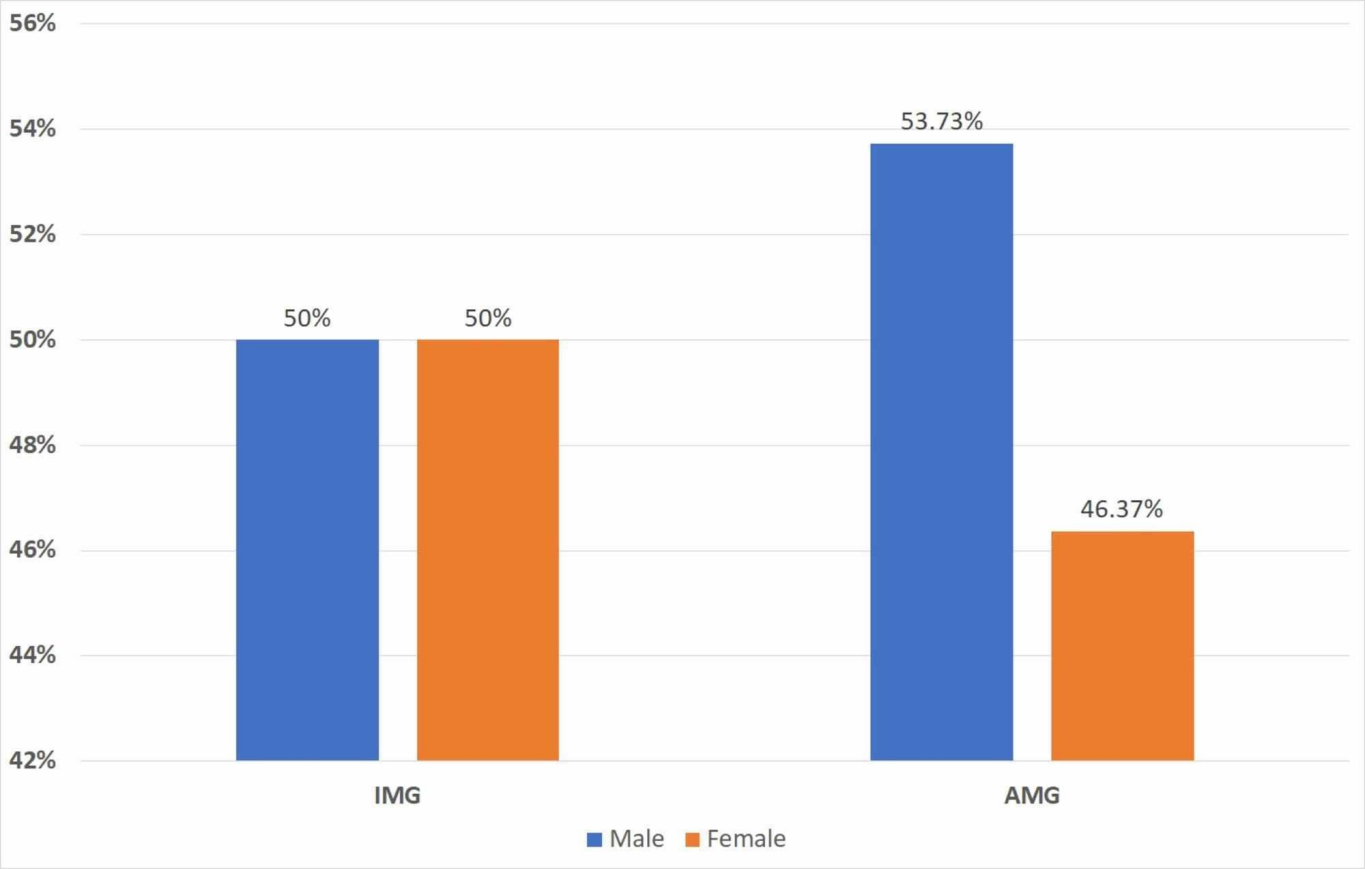
Gender compositions for international versus American medical graduate family medicine faculty IMG: international medical graduate; AMG: American medical graduate.

A relatively smaller percentage of females made up the American medical graduate (AMG) population (46.27%) compared to their international medical graduate (IMG) counterparts (50%) (Figure 1). 

Academic rank and leadership position

**Figure 2:**
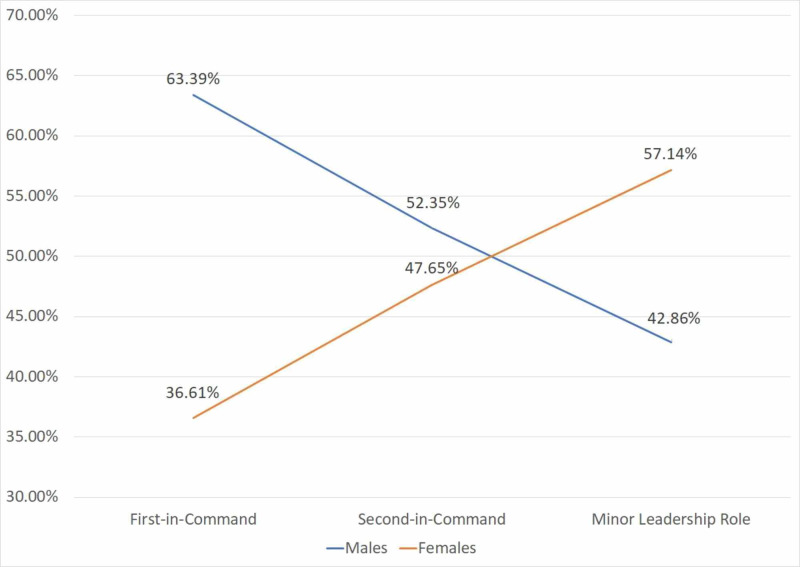
Gender compositions for different leadership positions First-in-Command: Heads, Chiefs, Chairperson, and Program Directors. Second-in-Command: Associate Directors & Associate/Deputy/Vice Chairperson. Minor Leadership Position: All other positions were labeled as "minor" leadership positions, which included positions that reported to someone that did not fall under any of the first- or second-in-command positions.

Academic rank and leadership position information was available for 2,887 and 1,702 faculty members, respectively (Table 1). Analysis by academic rank included sample sizes of 1,900 assistant professors, 575 associate professors, and 412 full professors. Analysis by leadership position included 1,008 first-in-command, 680 second-in-command, and 14 in minor leadership roles. In terms of academic rank, females comprised the minority in all positions: 49.8% (n=947) assistant, 46.8% (n=269) associate, and 41.5% (n=171) full professors (Figure 2, Table 1). As for leadership position, the female composition progressively declined as the leadership position rose: 57.14% (n=8) in minor leadership roles, 47.65% (n=324) second-in-command, and 36.61% (n=369) first-in-command (Figure 2, Table 1). 

Publication productivity

**Figure 3:**
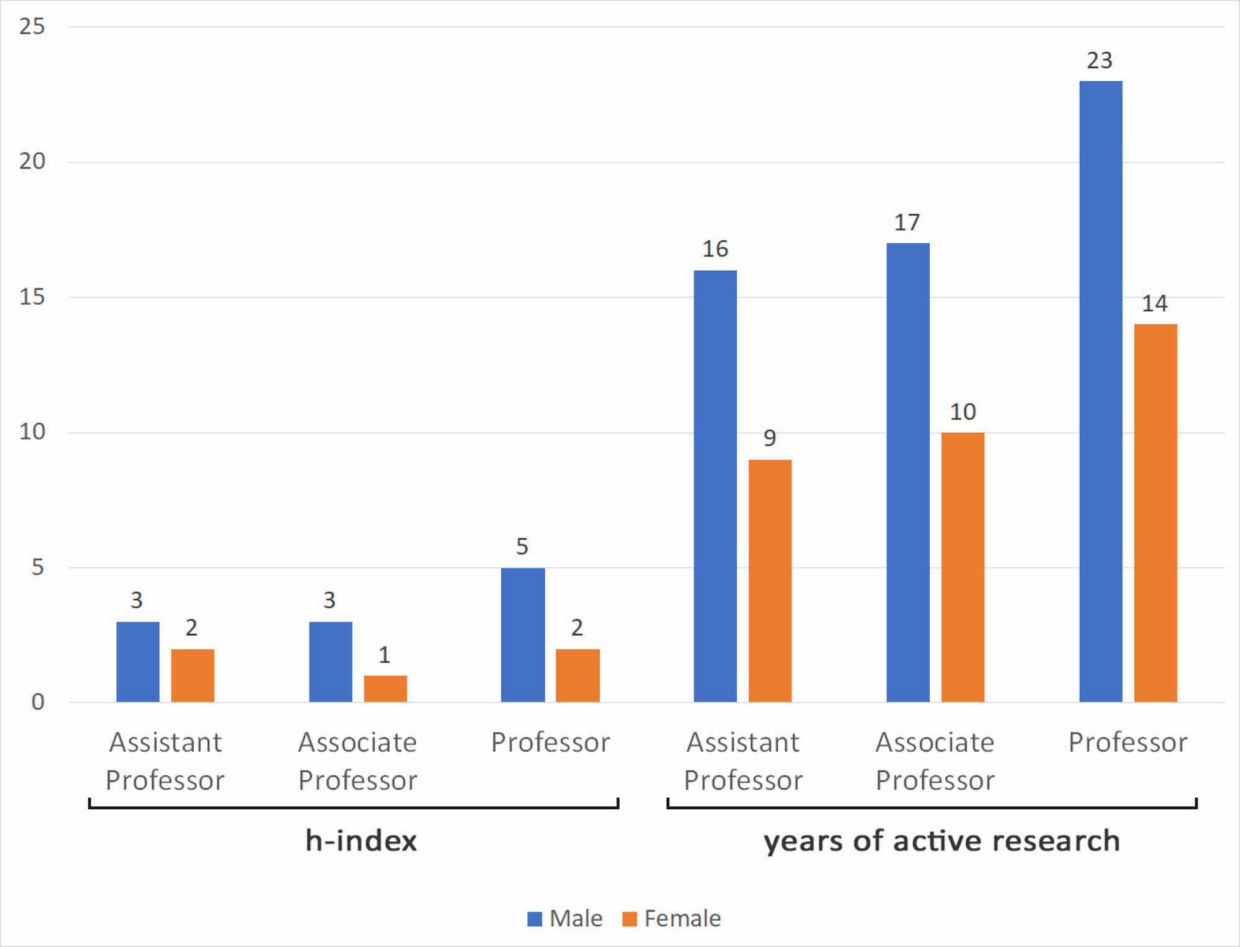
Distribution of h-index and years of active research across academic ranks and gender The values represent the median.

**Figure 4:**
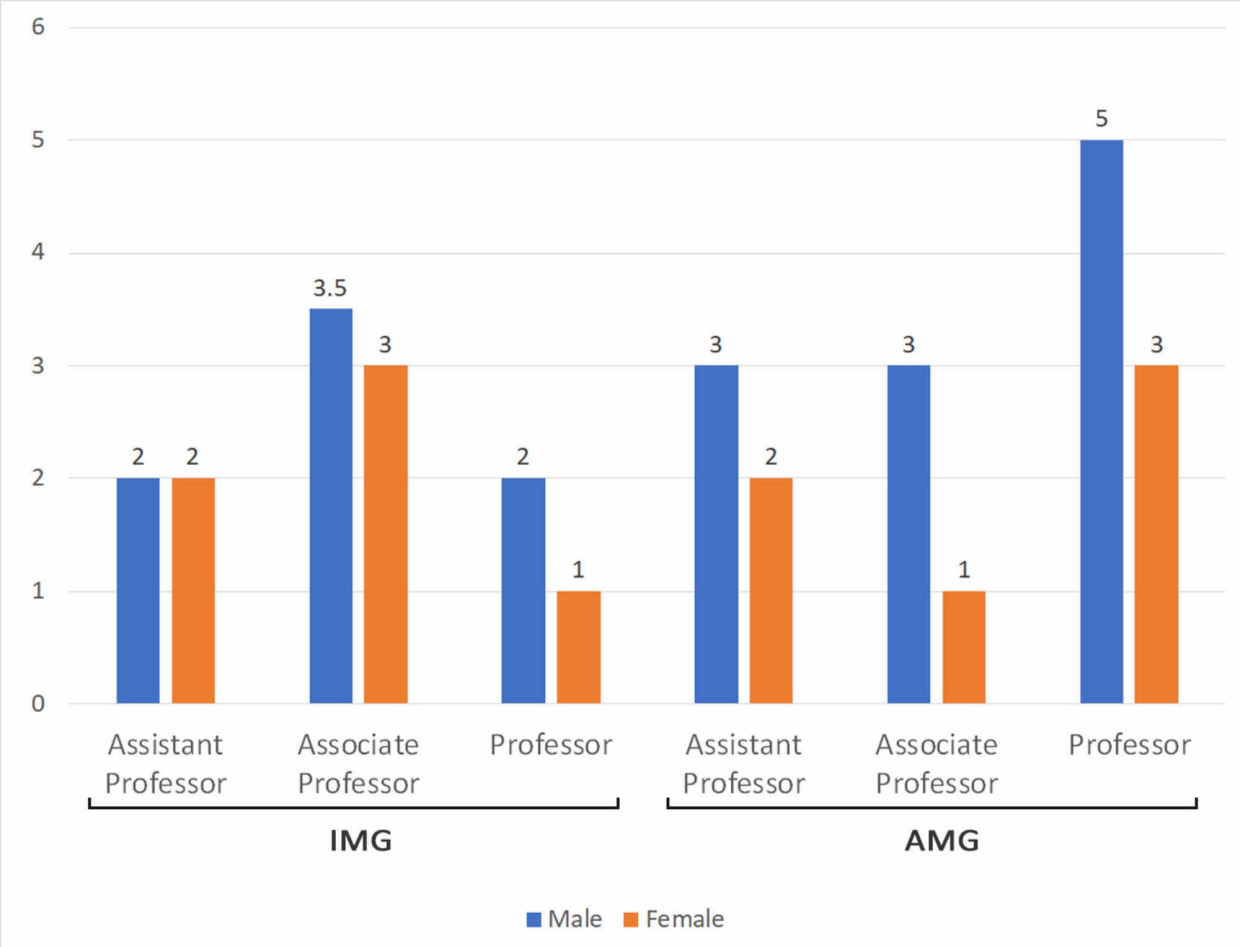
Comparison of the distribution of h-index across international and American medical graduates The values represent the median h-index. IMG: international medical graduate; AMG: American medical graduate.

The author metrics (number of citations, publications, h-index, and active years of research) for the family medicine faculty are presented in terms of the median and range (Table 2). Females had a lower number of years of active research compared to their male counterparts (p=0.008, Figure 3, Table 2). Women demonstrated lower median h-index across all academic ranks among both AMGs and IMGs, except in IMG assistant professor position in which there was no gender disparity (Figure 4, Table 2). There was no difference in overall h-index in women between AMGs and IMGs. However, IMGs had overall higher h-indices than their AMG associate professor counterparts.

**Table 2:** Research productivity metrics of the family medicine faculty

	Male Median (Range)	Female Median (Range)
Publications		
Assistant Professors	3 (1–204)	2 (1–382)
Associate Professors	4 (1–243)	3 (1–74)
Professors	4 (1–302)	3 (1–141)
Citations		
Assistant Professors	40 (0–31657)	14 (0–14457)
Associate Professors	48 (0–44957)	17 (0–3699)
Professors	101.5 (0–52113)	28 (0–6744)
H Index		
Assistant Professors	3 (0–85)	2 (0–67)
Associate Professors	3 (0–79)	1 (0–27)
Professors	5 (0–100)	2 (0–38)
Years of Active Research		
Assistant Professors	16 (0–112)	9 (0–72)
Associate Professor	17 (1–62)	10 (1–46)
Professors	23 (0–68)	14 (1–40)

Model creation protocol

Variables that were significant on univariate regression were gender (p=0.048), publications (p=0.02), citations (p=0.04), years of active research (p=0.008), and country of graduation. Academic rank and leadership rank were insignificant. The above significant variables were selected for inclusion into multivariable linear regression analysis. Academic and leadership ranks were dropped from the model as they were insignificant.

The multivariable analysis supported the inclusion of gender, citations, publications, and academic rank in the preliminary model (p<0.05). There was no multicollinearity observed using a correlation coefficient of 0.8. No significant interactions were found in the main effects in the model. None of the following were confounders for h index: academic rank, publications, and citations.

Prediction Equation Model

y(x) = β0 + β1 (Female) + β2 (Publications) + β3 (Citations) + β4 (Country of Graduation) + β5 (Years of Active Research)

This prediction equation for h-index as the primary outcome accounted for major variability in the model as adjusted R square was 0.80. The F test was 1741.2 and p-value was ≤0.001.

## Discussion

Gender disparity is particularly important in the domain of AFM, where the tone of its leadership is integral in bringing about meaningful and impactful change for the health of the populations that they take care of. Previous studies support our findings that women in academics sit in less than half of AFM faculty positions [3,4]. Increasing seniority in professorship was similarly associated with a widened disparity as fewer women sat in first command leadership roles [19]. The gap between women and their gender counterparts becomes apparent in the context of slower career advancements due to barriers to promotion, lower financial compensation, and many other reasons [20,21]. Controlling for specialty, seniority, hours worked, publications, and grants, this compensation disparity was, in fact, more significant at higher-level leadership roles [21].

Keeping this in mind, it comes to no surprise that instead of taking on more leadership roles, women choose to do the opposite: to leave their faculty position [22]. The contributing factors that lead to a lack of women in senior and leadership positions remain equivocal, but the need to decrease women faculty attrition rates is clear. The good news is: factors that contribute to poor faculty retention were found to be amendable to change and integral to retaining high-quality faculty and lowering costs associated with high attrition rates [22]. Understanding the issues that contribute to the differential treatment of women holds the potential for improvement of the academic medicine setting.

Previous studies demonstrated that an h-index of 10 is a reliable metric to determine the likelihood of receiving funding from the National Institutes of Health [23]. Since future academic success can be measured through research productivity, we propose regular monitoring of salaries and tracking of disparities [23]. Our study formulated a prediction equation model on factors that impact h-index: gender, publications, citations, country of medical school graduation, and years of active research. We believe this formula will assist with the monitoring of disparity. Results reveal that women faculty had lower academic productivity when measured by the median number of citations, publications, number of years of active research and h indices. Specifically for research development, prior studies demonstrated a lack of mentoring, research capacity, funding, and infrastructure as reasons for the observed discrepancy [24].

Our study provided a cross-sectional overview of the current state of gender disparity in AFM. We propose developing faculty recruitment and retention programs that focus on the variables suggested by our prediction model [25]. The first step is to obtain faculty input on how to improve retention as this has been shown to strengthen faculty diversity [26]. Women should be actively recruited into the faculty and provided with frequent mentorship and networking opportunities to increase the likelihood of promotion [27]. The creation of inclusive environments requires the removal of biases in policies and procedures. Our results also demonstrated a few differences in AMGs and IMGs. IMGs demonstrated no gender disparity in the composition of women in AFM and assistant professor positions. In fact, IMGs overall had higher h indices than their AMG associate professor counterparts. As no previous studies have been done on this population, this only provides a preliminary look at the current state and we propose hypotheses such as recruitment of IMGs favoring those with strong research backgrounds or interest in academic positions.

In addition to monitoring, incentives for academic engagement and research activities can also promote productivity [28]. Publications and citations may be increased by providing focused support on clinical, teaching, and research skills [29]. However, it is not that women lack the skills, it is the systematic discrimination despite having the same skills. In addition to recruitment, retention should be a key issue to address through decreasing prejudice and improving the gender wage gap. Depending on the needs of the individual, programs can also consider reducing clinical and administrative expectations to facilitate scholarly activities that lead to promotion. Other factors that might provide more time to be allocated to research include addressing subtle discrimination and providing assistance with family responsibilities such as child-rearing [29,30]. Academic centers now propose multiple tools for intervention, and the hope is that our prediction model can stand as a framework for measuring changes post-intervention.

Limitations

Our study has its share of limitations including its cross-sectional nature, which only provides a snapshot in time and does not provide a longitudinal history. As information is from websites, it is possible it is not up to date. Nonetheless, it can be effectively used to observe the trends in gender disparity. In addition, the data obtained only included full-time faculty and excluded part-time academics, which could impact the number of women faculty included in this study. In fact, the remaining variability in the model could be explained by variables such as full-time versus part-time employment, as well as years of employment and contract versus tenure positions. However, this was beyond the scope of our current project, as we used the data that were publicly available.

Another consideration is that often authors publish under different names. This is important in several scenarios such as when an individual, regardless of gender or sexual orientation changes their name after marriage or divorce, takes their spouses surname and creates a hyphenated name or a transgender that may change their name. When these name changes are taken into consideration, it shows that the number of publications and h-index for women faculty may be erroneously underestimated. The inclusion or exclusion of middle initials can also contribute to the miscalculations of academic productivity.

Lastly, we were unable to account for non-binary gender identities due to the design of this study. Future studies can overcome this limitation by conducting a survey (i.e. self-reported gender) instead of using publicly available data. Furthermore, the use of a survey would allow for the collection of additional information such as marital status and whether or not an individual has children.

## Conclusions

The composition of AFM faculty members included in this study demonstrated the extent of gender disparity and potential contributing factors. Inclusivity initiatives and policies to tackle the issue of female retention, promotion, and recruitment need to be further explored.
